# Light‐induced local gene expression in primary chick cell culture system

**DOI:** 10.1111/dgd.12721

**Published:** 2021-04-22

**Authors:** Keiichi Kitajima, Naofumi Kawahira, Sang‐Woo Lee, Koji Tamura, Yoshihiro Morishita, Daisuke Ohtsuka

**Affiliations:** ^1^ Department of Ecological Developmental Adaptability Life Sciences Graduate School of Life Sciences Tohoku University Sendai Japan; ^2^ Research Fellow (DC2) of Japan Society for the Promotion of Science Tokyo Japan; ^3^ Laboratory for Developmental Morphogeometry RIKEN Center for Biosystems Dynamics Research Kobe Japan; ^4^ Department of Anatomy and Cell Biology Graduate School of Medicine Kyoto University Kyoto Japan; ^5^ Precursory Research for Embryonic Science and Technology (PRESTO) Program Japan Science and Technology Agency Kawaguchi Japan

**Keywords:** limb mesenchyme, live imaging, optogenetics, primary cell culture, *Sox9*

## Abstract

The ability to manipulate gene expression at a specific region in a tissue or cell culture system is critical for analysis of target gene function. For chick embryos/cells, several gene introduction/induction methods have been established such as those involving retrovirus, electroporation, sonoporation, and lipofection. However, these methods have limitations in the accurate induction of localized gene expression. Here we demonstrate the effective application of a recently developed light‐dependent gene expression induction system (LightOn system) using the *Neurospora crassa* photoreceptor Vivid fused with a Gal4 DNA binding domain and p65 activation domain (GAVPO) that alters its activity in response to light stimulus in a primary chicken cell culture system. We show that the gene expression level and induction specificity in this system are strongly dependent on the light irradiation conditions. Especially, the irradiation interval is an important parameter for modulating gene expression; for shorter time intervals, higher induction specificity can be achieved. Further, by adjusting light irradiation conditions, the expression level in primary chicken cells can be regulated in a multiple step manner, in contrast to the binary expression seen for gene disruption or introduction (i.e., null or overexpression). This result indicates that the light‐dependent expression control method can be a useful technique in chick models to examine how gene function is affected by gradual changes in gene expression levels. We applied this light induction system to regulate *Sox9* expression in cultures of chick limb mesenchyme cells and showed that induced SOX9 protein could modulate expression of downstream genes.

## Graphical abstract

The ability to manipulate gene expression at a specific region in a tissue or cell culture system is critical for analysis of target gene function in the process of development. In this study, we tested whether the LightOn system, recently developed light‐dependent gene expression induction system, works in embryonic chicken cells. Using Sox9 gene as a target, we succeeded in inducing its own expression and upregulating the expressions of its downstream genes, demonstrating that this system will be a good tool for analysis of gene function in chicken models.

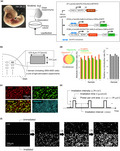

## 1 INTRODUCTION

The ability to induce or suppress expression of a given gene at a specific location in a tissue and with specific timing is essential for analysis of gene function in developmental processes. Various methods for gene introduction have been established for different model organisms. For chickens, a well‐established animal model for developmental biology studies, different gene introduction approaches are available including methods that involve retroviruses, electroporation, sonoporation or lipofection (Bollérot et al., 2006; Decastro et al., 2006; Morgan & Feketet, 1996; Muramatsu et al., 1997; Ohta et al., 2008; Takase & Takahashi, 2019). Among these approaches, electroporation methods are the most commonly used and allow a target gene to be introduced and expressed locally and temporally by limiting the location of the DNA solution and electrode. However, in practice, accurate spatial and temporal control of gene expression levels is difficult due to expansion of tissue arising from cell proliferation and limitations in specifying the timing of gene expression using regulatory sequences such as promoters. Such limitations in controlling the timing of gene expression induction can be overcome by combining gene introduction methods with gene induction techniques such as the Tet‐on system, which is driven by chemical treatment, but a technique to increase the accuracy of expression localization has not yet been established (Hou et al., 2011; Nakamura & Funahashi, 2013; Sato et al., 2007; Sauka‐Spengler & Barembaum, 2008).

In recent years, a technique for manipulating cell functions using proteins that undergo light‐dependent structural changes has been developed. This technology was first used to control neuronal activity by spatiotemporally manipulating the state of channel proteins (Deisseroth et al., 2006). More recently, this technique was applied to the structural transformation of transcription factors, which allowed control of gene expression in a light‐dependent manner. The LightOn system is a light‐dependent technique that uses the GAL4 upstream activating sequence (UAS) and the artificial protein GAVPO that comprises three factors, the GAL4 DNA binding domain, the VIVID protein that undergoes light‐dependent dimerization, and the transcription activation domain of the transcription factor P65. Successful use of this system for light‐dependent temporal and spatial control of gene expression has been reported for HEK293 cells and mice (Wang et al., 2012). In addition, Imayoshi et al. (2013) used this technique to show that light–induced oscillatory Ascl1 expression triggers proliferation of neural progenitor cells, whereas light‐induced sustained Ascl1 expression promotes differentiation into nerve cells (Imayoshi et al., 2013). These results indicate that a technique that allows precise spatiotemporal control of gene expression in cells is useful to understand developmental phenomena that could be affected by expression position and/or timing.

As a potential technique for increasing the accuracy of expression localization for studies using chickens, here we introduced the LightOn system into embryonic chicken cells through the use of a primary culture system of limb mesenchymal cells and tested its performance by investigating the relationship between light stimulation conditions and specificity of gene expression induction. We confirmed that with the selection of appropriate light stimulation conditions we could achieve sufficiently high induction specificity wherein the expression of the target gene was induced in most cells with the LightOn system. We also tested the light‐dependent expression of *Sox9*, a key transcription factor for cartilage differentiation, and confirmed that the induced SOX9 proteins are functional in that some genes downstream of *Sox9* were locally activated. These results demonstrate that the LightOn system works efficiently in chicken cells and will be a good tool for analysis of gene function in chicken models.

## 2 MATERIALS AND METHODS

### 2.1 Embryos

Fertilized chicken eggs (Inoue Egg Farm) were incubated in a humidified incubator at 38°C until the embryos reached the appropriate stage (Hamburger & Hamilton, 1951).

### 2.2 Collection of embryos and primary cell culture

Chick primary cell cultures were performed as previously reported (Hattori & Ide, 1984) with the following modifications. Hindlimb buds from Stage 26–27 chicken embryos were excised and washed in cold Ca^2+^‐ and Mg^2+^‐free Tyrode solution (CMF). They were then transferred to cold CMF with 0.5% trypsin (Difco, 1:250), and incubated at 4°C for 30 min. After this treatment, the loosened ectoderms were peeled off with a tungsten needle. The denuded mesoderms were collected and incubated in CMF at 37°C for 30 min, and the softened tissues were dissociated into a cell suspension by gentle pipetting in 1% Fetal Bovine Serum (FBS) (Gibco, 26140087)/Ham's F12 (Sigma‐Aldrich). Dissociated limb bud cells were diluted with 1%FBS/Ham's F12 to 8 × 10^5^ cells/mL. Small stainless‐steel columns (penicillin cup, 6 mm ϕ) were fixed with silicone grease on a plastic dish (3002, Falcon) and 10 µl of cell suspension (2.4 × 10^5^ cells) was poured into each well. The cells were allowed to adhere for 4 hr at 37°C, 5% CO_2_ before the columns were removed. The cultures were then fed with 4 ml 1% FBS/Ham's F12.

### 2.3 Construction of plasmids

**FIGURE 1:**
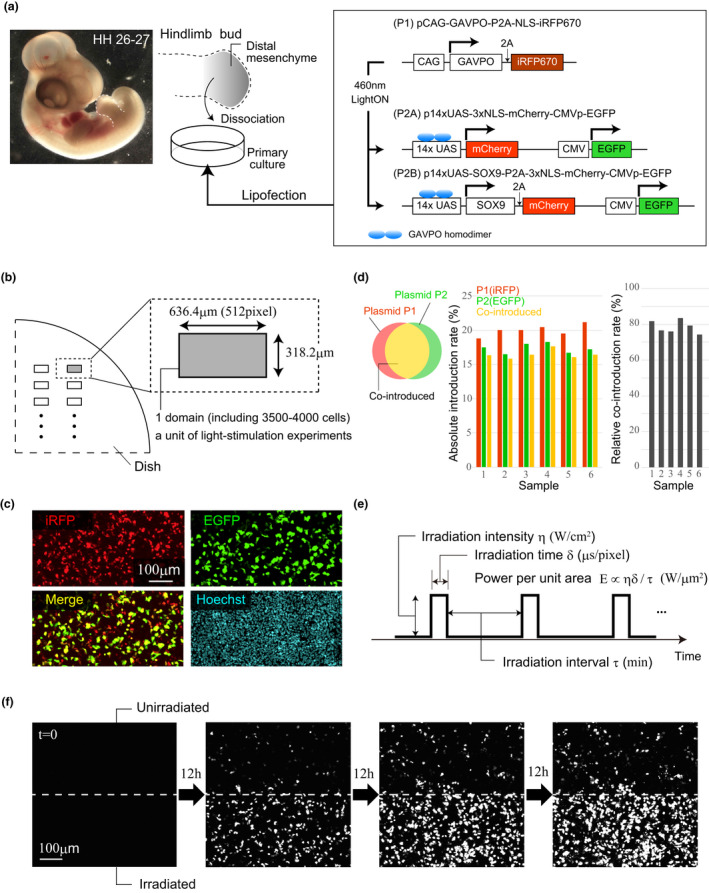
Design of DNA constructs for light‐induced gene expression. (a) DNA constructs designed for local induction of gene expression by light stimulation. Plasmid P1 carries the GAVPO gene and iRFP670 as a reporter. Plasmids P2A and P2B both contain constitutively‐expressed EGFP as a reporter, and upstream activating sequences UAS to which light‐activated GAVPO protein binds. P2A has only mCherry as a GAVPO‐UAS target gene, whereas P2B has both mCherry and *Sox9*. (b) Unit for analysis in the light stimulation experiment. For local induction of gene expression, the light was irradiated to the several rectangular regions in the culture system. (c) Representative fluorescence images of EGFP and iRFP in primary cell cultures co‐transfected with plasmids P1 and P2. Nuclei are stained with Hoechst33342. (d) Absolute introduction rate and relative co‐introduction rate of plasmids (P1 and P2A) quantified for six regions after 10 hr of culture. (e) Laser irradiation parameters for light‐induced gene expression. (f) Typical temporal changes in mCherry expression for unirradiated (top) and irradiated (bottom) regions. The white broken lines show the boundary of both regions

To generate LightOn system constructs (Wang et al., 2012), we initially designed (a) a plasmid to express the GAVPO protein under control of the CAG promoter (pCAG‐GAVPO‐P2A‐Lyn‐mCherry) and (b) a plasmid to express SOX9 protein that contained upstream activating sequences (UAS) (p14xUAS‐SOX9‐P2A‐3xNLS‐mCherry‐CMVp‐EGFP), termed P2B (Figure 1a). These plasmids were synthesized by VectorBuilder Japan (Kanagawa). However, since distinguishing cells containing either or both of these two plasmids based only on membrane and nuclear mCherry signals was difficult, we replaced the Lyn‐mCherry in plasmid (a) with the NLS‐iRFP670 gene that encodes a fluorescent protein with a nuclear localization signal peptide to generate pCAG‐GAVPO‐P2A‐NLS‐iRFP670, termed plasmid P1 (Figure 1a). To examine the dependence of expression induction efficiency on light stimulation conditions, we made the plasmid P2A (p14xUAS‐3xNLS‐mCherry‐CMVp‐EGFP; Figure 1a) by deleting the *Sox9* gene in plasmid (b) using a PrimeSTAR Mutagenesis Basal Kit (Takara). The GenBank sequence for chicken *Sox9* was used (Accession number: 374148). Requests for plasmids should be directed to and will be fulfilled by the corresponding author.

### 2.4 Transfection

HilyMax (H357; Dojindo Molecular Technologies, Inc.) was used for plasmid DNA transfection. The plasmid DNA solution was diluted with Ham's F12 before mixing with the transfection reagent (HilyMax reagent) to yield a final DNA concentration of 1.7 ng/μl (1:6 plasmid DNA [ng] : transfection reagent). The mixture was incubated at room temperature for 15 min to allow lipid–DNA complexes to form. Using a micropipette, 30 μl of the lipid‐DNA complex solution (DNA sol.) was added to the culture medium (Ham's F12) and the mixture (DNA sol. and Ham's F12) was incubated at 37°C for 3 hr before the culture medium was replaced with 1%FBS/Ham's F12. Experiments were performed under dark conditions to avoid major leakage of expression that could occur upon exposure to typical room illumination. Cells at St. 26–27 were used based on their higher transfection efficiency compared to cells at earlier stages (Figure S1).

### 2.5 Immunocytochemistry of primary cell cultures

Primary cell cultures were fixed for 15 min at room temperature with 3% formaldehyde/PBS. The fixed cultures were washed and permeabilized with 0.1% Triton X‐100/PBS at room temperature for 10 min, incubated with primary antibodies diluted in PBS at room temperature for 1 hr, washed with PBS, and then incubated with regular secondary antibodies conjugated to Alexa 405 (Life Technologies) for 1 hr at room temperature. The following primary and secondary antibodies were used: Anti‐SOX9 antibody (Merck Millipore, AB5535), Anti‐SOX5 antibody (Abcam, ab94396), Anti‐SOX6 antibody (Abcam, ab30455), Anti‐p21 antibody (Cell Signaling Technology, 2946), Goat anti‐Rabbit IgG H + L Alexa Fluor 405 (Thermo Fisher, A‐31556), Goat anti‐mouse IgG H + L Alexa Fluor 405 (Abcam, ab175660).

### 2.6 EdU assay

EdU (Click‐iT™ EdU Cell Proliferation Kit for Imaging, Alexa Fluor™ 488 dye, Thermo Fisher, C10337) was diluted in cell culture medium to make a 10 µM labeling solution that was added to the culture dish 1.5 days after transfection. The cells were then incubated for 6 hr, and fixed for 30 min at room temperature with 3% formaldehyde/PBS. The fixed cultures were washed and permeabilized with 0.1% TritonX‐100/PBS at room temperature for 10 min. To suppress crosstalk caused by EGFP fluorescence signals, the fixed cultures were incubated in 1 M HCl for 20 min, neutralized with 0.1 M sodium borate buffer pH 8.5 for 30 min, washed with PBS, incubated with Click‐iT reaction cocktail at room temperature for 30 min, and washed with PBS.

### 2.7 Imaging and optical stimulation

Live imaging was performed using a confocal inverted microscope (IX83, OLYMPUS) equipped with an Olympus UPLSAPO 20×/0.75 dry objective combined with a stage‐top incubation chamber (IX3WX; TokaiHit). For high resolution live cell imaging, stacks of optical section images (40 slices, 512 × 512 or 512 × 256 pixels for the x‐y plane and 2 µm for the *z*‐axis step) were acquired at 10, 20 or 60 min intervals over 4 days. For light irradiation control, we used Olympus FV31S‐SW software, which allows specification of the range, intensity, and interval of irradiation by LSM stimulation command. A 405 nm laser was used. The details of the irradiation conditions are shown in Table 1 and Figure 1e. A cell proliferation assay showed that, at the irradiation intensity needed to induce gene expression, the laser had little effect on the cell proliferation rate and thus was unlikely to cause cell damage (Figure S1).

**TABLE 1:** Light irradiation conditions tested in this study

Condition	η	δ	τ	E (× 10^‐14^)	*N* (domains)
0	0	0	0	0	16
1	0.022	8	20	0.143	4
2	0.0077	24	20	0.154	3
3	0.022	24	60	0.143	3
4	0.071	8	60	0.158	3
5	0.0077	72	60	0.154	3
6	0.022	8	60	0.0478	4
7	0.022	8	10	0.287	4
8	0.0077	8	20	0.0513	6
9	0.0077	24	60	0.0513	3

### 2.8 Image analysis

**FIGURE 2:**
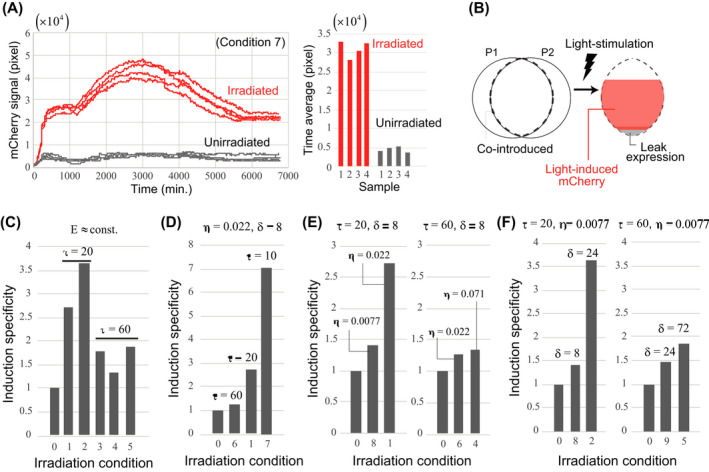
Light stimulation conditions and induction specificity of gene expression. (a) Time series (left) and time average (right) of the mCherry signal in each region irradiated under Condition 7 and unirradiated. (b) Observed mCherry signal includes light‐induced and leak expression. (c) Comparison of the induction specificity, S, between conditions in which the energy per unit area per unit time, E, is almost equal. (d) Dependence of induction specificity on τ for a fixed set of η and δ. (e) (f) Dependence of induction specificity on η and δ for different irradiation intervals, τ

**FIGURE 3:**
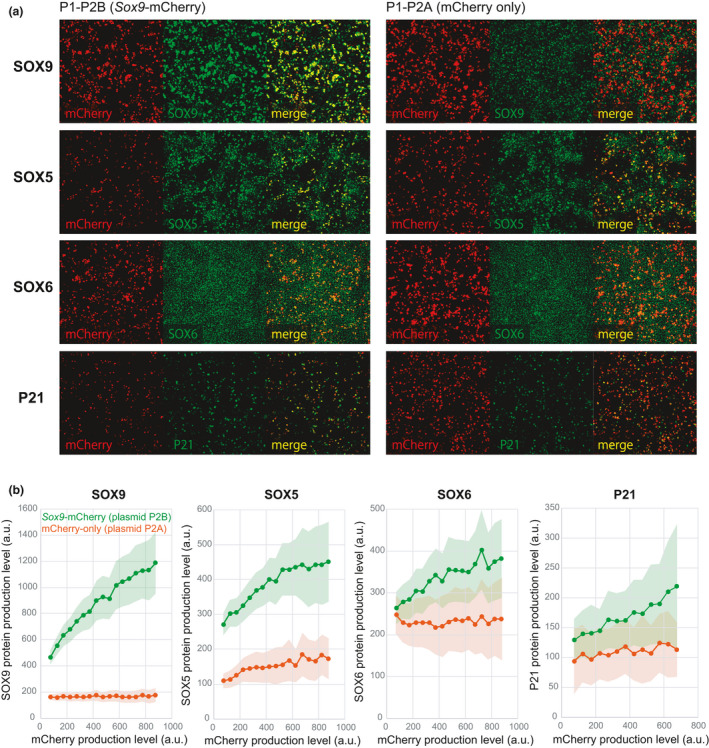
Induction of expression of *Sox9* and its downstream genes by light stimulation. (a) Representative images of antibody staining for SOX9, SOX5, SOX6 and P21 in cultured cells co‐transfected with plasmids P1 and P2B (i.e., *Sox9*‐induced) or P1 and P2A (i.e., control). (b) Dependence of expression levels of *Sox9* and its downstream genes on mCherry expression level for culture systems with P1‐P2B (green) and P1‐P2A (red). Points indicate the mean expression levels and light colors indicate standard deviations (statistics were calculated over pixels for each mCherry signal bin)

Fiji software was used for all image analyses (see Figures 1d, f, 2a, c–f, 3b and S1). In the analysis, we first constructed Z projection images from the acquired 3D stack images using maximum projection methods, and the Z‐projected images were used for all analyses except for the cell proliferation assay (Figure S1). To quantify the efficiency of plasmid transfection (Figure 1d), we used manual and automatic cell counting. For manual counting, we used the “Cell Counter” plugin in the Fiji software (Schindelin et al., 2012). For automatic counting, we first applied a Gaussian filter (radius = 1pixel) to the Z‐projected images to remove noise. We then calculated the cell number from the filtered images using the “find Maxima” tool in Fiji. To count the number of cells carrying both plasmids (P1 and P2), we used images obtained by merging of the iRFP and GFP images for plasmids P1 and P2, respectively, and determined the total cell number after staining nuclei with Hoechst33342. To quantify the induction efficiency of gene expression by light stimulation (Figure 2), we measured the areas of fluorescent (mCherry) signals. To adjust the average brightness for the time series images, we used the “histogram matching” tool in Fiji, and binarized the processed images using the default Auto Threshold method. Subsequently, we applied the 2D median filter (radius = 1.5 pixels) to the binarized images to eliminate noise. The area of fluorescence signals was calculated from the resulting images. For quantitative comparison of SOX9, SOX5, SOX6 and P21 expression levels between culture systems transfected with P1‐P2B (*Sox9*‐mCherry) and P1‐P2A (mCherry only) (Figure 3b), clusters of signals that were substantially smaller than that of nuclei were first removed from the mCherry images. The removal threshold was determined visually and the same threshold was used for all images. Then, using the immuno‐staining images for SOX9, SOX5, SOX6 and P21, the mean and standard deviation of the protein production level for each gene over image pixels against mCherry expression level was plotted for each bin, where the bin size was set to be 50 (a.u.). In the cell proliferation assay (Figure S1b,e), the Z‐plane having the highest nuclear density was selected for the analysis. All the nuclei and EdU‐positive cells were detected using the Fiji plugin StarDist (Schmidt et al., 2018). In the comparison of transfection/co‐transfection rate of plasmids between samples from different developmental stages (Figure S1g), the iRFP positive cells were firstly detected using the StarDist plugin to evaluate the plasmid transfection rate and then co‐transfection rate was quantified as the ratio of the number of cells showing both iRFP and GFP expressions to that of iRFP positive cells.

### 2.9 Calculation of specificity of gene expression induction by light stimulation

Expression induction specificity (S) in a sample was calculated as the ratio of the average mCherry signal over time and sample for the irradiated region to that for the unirradiated region (see Figure 2c–f). The frequency of cells with mCherry expression in the unirradiated region can be estimated as the product of the number of cells in the region (*N*_cell_), rate of plasmid P1 and P2 co‐introduction (*f*_co_), and the leak expression rate (*f*_leak_), i.e., *N*_cell_ *f*_co_ *f*_leak_. The frequency of cells with mCherry expression in the irradiated area can be estimated as: *N*_cell_ *f*_co_ (1‐(1‐*f* *_leak_*)(1‐*f*_induce_)) using the induction rate *f*_induce_; note that cells not expressing mCherry are those without both leak and induction expressions and the frequency of those cells is given by (1–*f*_leak_)(1–*f*_induce_). The induction specificity S can then be expressed as:

$$S=1+\frac{f_{\text{induce}}}{f_{\text{leak}}}‐f_{\text{induce}}.$$

When *f*_leak_ is not large, $S\approx 1+f_{\text{induce}}/f_{\text{leak}}$ holds.

## 3 RESULTS

### 3.1 Design of a DNA construct to evaluate the LightOn system in chicken cells

To induce gene expression via the GAVPO protein by light stimulation in chicken cells, we first designed the plasmids P1, P2A and P2B. P1 carries the GAVPO gene and iRFP670 as a reporter to confirm successful introduction into cells (Figure 1a). P2A and P2B (referred to collectively as P2) both contain constitutively‐expressed EGFP as a reporter, and upstream activating sequences (UAS) to which light‐activated GAVPO protein binds. Since repeated UAS are known to be more responsive to activated GAVPO (Akitake et al., 2011), these plasmids carried 14× tandem UAS to promote higher expression levels. P2A has only 3xNLS‐mCherry (hereafter referred to as mCherry) as a GAVPO‐UAS target gene, whereas P2B has both mCherry and *Sox9* (Figure 1a). P2A was used to examine light stimulation conditions suitable for gene expression, and P2B was used to test the inducibility of functional proteins in a more biologically relevant context. We used primary limb bud mesenchymal cells isolated from chicken embryos because they are relatively readily available in large numbers. Furthermore, the use of a 2D culture system has the advantage that areas for light irradiation can be easily limited.

First, we examined the introduction efficiency of plasmid P1 and P2 (i.e., P2A or P2B). For quantification of gene expression, a 636.4 × 318.2 μm rectangular region that included several thousand cells served as a sample unit (Figure 1b). We confirmed through measurement of iRFP, EGFP, and Hoechst fluorescence in samples that the plasmids could be successfully introduced singly or co‐introduced into cells (Figure 1c). We measured the introduction efficiency in each region (Figure 1d). In our experimental settings, the absolute rate of plasmid co‐introduction was not high (15%–20%), but these rates were reproducible between samples. This high reproducibility of the introduction rate indicates that, in the following light stimulation experiments, the expression induction specificity can be approximately measured using the ratio of the number of mCherry‐expressing cells in irradiated and unirradiated regions. On the other hand, the relative rate of co‐introduction of both plasmids, i.e., the ratio of the number of cells having both plasmids compared to cells that had at least one or both plasmids, was high (75%–85%; Figure 1d). It should be noted that since the wavelength of the laser used to observe the GFP signal also affects GAVPO activation, GFP signals were measured to examine the introduction efficiency of plasmids, and were not monitored in subsequent analyses.

To confirm light‐induced target gene expression, the cell cultures were repeatedly irradiated with a laser having a wavelength of 405nm soon after initiation. Three parameters were used to control laser irradiation (Figure 1e): (a) irradiation intensity η (W/cm^2^), (b) irradiation time per unit area per one irradiation δ (us/pixel), and (c) irradiation time interval τ (min). The light‐induced expression level was monitored by the total number of pixels showing a mCherry signal. Under conditions of η = 0.022, δ = 8, τ = 10, target gene expression was significantly induced by light stimulation, although leak expression was also detected in the unirradiated region (Figure 1f and Movie S1). As will be seen in detail in the next section, the induction performance measured by the induction specificity varied greatly depending on the laser irradiation conditions. Note that, in our system, no mCherry expression was detected in control experiments in which either plasmid P1 or P2 was introduced alone.

### 3.2 Conditions for light stimulation and induction specificity of gene expression

Next, we investigated the dependency of expression induction specificity on light irradiation conditions by examining nine different conditions for which mCherry signals were measured simultaneously with light irradiation timing (Conditions 0–9, see Table 1). As an example, Figure 2a shows the time series (left) and time average (right) of the mCherry signal in each region irradiated using Condition 7 and control (i.e., unirradiated, Condition 0), where all regions for both conditions were included in the same culture dish. Regardless of the light stimulation conditions, we confirmed that the time average of the mCherry signal was sufficiently reproducible between samples within the same dish. The specificity of expression induction (denoted by S) by light irradiation was quantified as the ratio of the average of the mCherry signal over time and sample for the irradiated region to the unirradiated region. In principle, *S* values will be > 1 under any light stimulation conditions, and *S* = 1 corresponds to the leak expression level (Figure 2b).

We first compared the induction specificity (*S*) between conditions where the energy per unit area per unit time E (i.e., irradiance [η] × irradiation time [δ] ÷ irradiation interval [τ]) was almost equal (Condition 1–5) (Figure 2c). Importantly, the induction specificity differed even when the same amount of energy (E) was applied, indicating that *S* is not related simply to the amount of energy applied to the cells. Since *S* more clearly varied depending on the irradiation time interval (τ), we next examined the induction specificity when τ was changed for a certain irradiation time (δ) and irradiance (η) (i.e., comparing conditions 1, 6, and 7). We observed that *S* was strongly dependent on τ (Figure 2d). When τ = 10 min, target gene expression was induced in most cells in which plasmids P1 and P2 were co‐introduced. Furthermore, the irradiation time interval also affected the sensitivity of *S* to the other irradiation parameters, η and δ. An examination of the dependence of *S* on η and δ at irradiation intervals τ = 20 min and 60 min showed that at the shorter interval (τ = 20 min) the sensitivity of S to both irradiation parameters (i.e., η and δ) was high, whereas for the longer interval (τ = 60 min), the sensitivity to the parameters was substantially lower (Figure 2e,f). In comparing τ = 20 and τ = 60, the combination of δ and η values was chosen so that the energy E was almost the same.

Thus, the specificity of light‐induced gene expression has a nonlinear dependence on these different irradiation parameters. In particular, the irradiation interval τ was found to be a parameter that had a significant effect on the specificity of induction. Importantly, as reported in mammalian cell culture studies (Wang et al., 2012), adjusting the light irradiation conditions allowed multi‐step regulation of expression levels, which is in contrast to the binary expression achieved by gene disruption or introduction (i.e., null or excessive overexpression). This indicates that the light induction system can also be a useful tool in chick studies to examine the dependence of gene functions on expression levels in a gradual, rather than binary, manner.

In terms of the time scale for induction in our system, fluorescence from reporter proteins was detectable within 1–2 hr after light stimulation. Furthermore, when comparing conditions 2 and 9, which have different irradiation intervals (τ) and an identical energy pulse (δ × η), sufficient induction of expression was maintained for condition 2 that has a 20‐min irradiation interval, but for the 60‐min interval used for condition 9, the expression was closer to the leak level. This result suggests that the time scale of conformational change of the GAVPO protein from the on to off state is on the order of tens of minutes. These results are also consistent with a previous report involving human HEK293 cells (Wang et al., 2012) wherein the time scale for switching between on and off states was estimated to be 1–2 hr based on measurements of mRNA abundance.

### 3.3 An application: expression induction of functional SOX9 protein by light stimulation

To demonstrate that the LightOn system can induce production of functional proteins, we next induced expression of *Sox*9, a key transcription factor for cartilage differentiation in limb mesenchyme (Akiyama et al., 2002, Akiyama et al., 2002, 2007, Bi et al., 1999, Bi et al., 2001), using a primary culture system (Hattori & Ide, 1984). In addition to *Sox9* expression, we also observed expression of the downstream targets of *Sox9*: *Sox5*, *Sox6* and *cyclin dependent kinase inhibitor 1A* (*p21/CIP*; hereafter referred to as *p21*) (Akiyama et al., 2002, Passeron et al., 2009).

Culture systems co‐transfected with plasmids P1 and P2A (control) or P1 and P2B (*Sox9*‐induced) were stimulated by light under Condition 7 that showed the highest specificity (Table 1) for 3.5 days. The cells were then fixed and stained with antibody for SOX9, SOX5, SOX6 or P21 (Figure 3a). For all samples, the relationship between the expression level of each of these genes and that of mCherry was examined at each mCherry‐positive pixel after removal of noise from the mCherry images (Figure 3b and Materials and Methods). In the culture system co‐transfected with P1‐P2A, we first confirmed that, regardless of mCherry level, the *Sox9* expression level was almost constant at each pixel, and had a value that corresponded to the light induction‐independent expression level originating from the limb mesenchyme primary culture system (Figure 3b and see also Materials and Methods). On the other hand, in the culture system with P1‐P2B, mCherry and *Sox9* expression levels showed an almost linear relationship, indicating that production of SOX9 proteins was properly induced by light stimulation. Similarly, expression levels of downstream genes of *Sox9* were significantly higher in the culture system with P1‐P2B and showed a clear linear relationship with the mCherry expression level (Figure 3b). These results show that functional *Sox9* expression can be induced by light stimulation.

## 4 DISCUSSION

In this study, we demonstrated that the LightOn system, in which light stimulation is used to induce gene expression, is effective in embryonic limb mesenchymal cells from chickens. Chicken embryos have long been used as a model for developmental biology because of their ease of manipulation. The DNA constructs we made for this study will be applicable to research using embryos. For example, *Sox9* is known to be involved in neural crest cell differentiation and movement (Sakai et al., 2006). Since the dorsal region of the neural tube where the neural crest cells first appear is close to the surface and can be readily irradiated with light under a microscope, we can induce *Sox9* expression for analysis of gene function at higher spatial resolution than was previously possible. Developing limbs could also be a potential target for this application. Hox genes are involved in limb spatial patterning (Zakany & Duboule, 2007); for example, Hoxa13 and Hoxa11 are well known to be typical marker genes for the autopod and zeugopod regions, respectively, and Hoxd13 is expressed in the interdigital regions at the autopod stage and is thought to be involved in digit identity determination (Dollé et al., 1989; Fromental‐Ramain et al., 1996; Suzuki et al., 2004). Thus, with the LightOn system, we could manipulate the skeletal proportion/identity by spatiotemporally regulating their expression levels. Due to the thickness of limb tissue, however, the LightOn system would need to be combined with another (invasive) device, such as optical fibers (Rivnay et al., 2017), in order to photo‐stimulate deeper tissue regions. More recently, optogenetic techniques have also been used as a tool to manipulate cellular behavior during morphogenesis. For example, apical constriction could be induced through light‐stimuli‐dependent localization of Rho‐GEF to the plasma membrane using light‐induced CRY2‐CIBN binding system in specific cell populations (Krueger et al., 2019). The LightOn system could also be used to alter cellular mechanical states by inducing expression of active forms of RhoA and ROCK that are involved in the Rho signaling pathway, which would be useful for study of morphogenetic mechanisms.

To date, several other systems to induce gene expression by light stimulation have been developed (Krueger et al., 2019; Polesskaya et al., 2018). Each of these systems has advantages and disadvantages. For example, the LightOn system has an advantage in that it requires only weak light stimulation to induce gene expression, although expression can be leaky and occur even in the absence of light stimulation (Ma et al., 2013, Wang et al., 2012). This leakage problem can be addressed through use of the tet‐on system (Yamada et al., 2018). Further, cytotoxicity was also reported for GAVPO expression in zebrafish, and such toxicity could be suppressed by modifying the transcription activation domain (the modified construct was referred to as TAEL (Reade et al., 2017)). In this study, we observed no significant cytotoxicity in that there was little change in the cell proliferation rate upon gene induction (Figure S1), but we did observe leakage expression in the absence of light stimulation.

Bobick et al. introduced plasmids carrying the *Sox9* gene into mesenchymal cells isolated from chick wing buds and the cells were grown in primary cell culture (Bobick et al., 2014). On Day 3 of culture they observed increased expression of collagen type II expression in cells to which the *Sox9* plasmid was introduced relative to control cells. They reported that *Sox9* overexpression reduced the area of the inter‐nodule region, which led to the formation of a spatially uniform cartilage sheet structure. In our study, light‐induced *Sox9* gene expression could increase the expression of downstream genes (*Sox5*, *Sox6*, and *p21*), but we saw no clear change in global patterns of cartilage formation. One possible reason for this outcome is that the gene introduction efficiency was lower than that in previous studies (15%–20% in this study versus. 70% in Bobick et al. (2014)). Thus, improvements in the efficiency with which the DNA constructs are introduced are needed. One approach to increase the introduction efficiency is optimization of reagents for lipofection, particularly given that gene introduction efficiency was reported to vary based on the type of lipofection reagent used (Takase & Takahashi, 2019). Gene introduction methods using RCAS virus or electroporation might also improve introduction efficiency.

We found that the efficiency of gene expression induction varies depending on the light stimulation conditions (e.g., irradiation intensity, irradiation time per single irradiation, irradiation time interval) even if the energy per unit time received by each cell is almost the same. The exact reason for this finding is unknown, but it could be related to the time scale of activation and inactivation of the VIVID protein LOV domain. In *in vitro* measurements, EL222, a protein having an LOV domain similar to that of the VIVID protein, is activated within micro‐to‐milliseconds by light stimulation and was inactivated from within seconds to hours (Chen et al., 2007, Harper et al., 2004, Kennis et al., 2003, Zoltowski et al., 2011). As such, expanded understanding of the activation dynamics of proteins carrying a LOV domain could contribute to optimization of light stimulation conditions.

## Supporting information

Fig S1

Movie S1
